# Efficacy of high-dose vs. low-dose intravitreal ganciclovir for cytomegalovirus retinitis: a systematic review and meta-analysis

**DOI:** 10.12701/jyms.2026.43.13

**Published:** 2026-01-14

**Authors:** Seongyong Jeong, Areum Jeong, Jae Rock Do, Yong Koo Kang, Min Sagong

**Affiliations:** 1 Department of Ophthalmology, Yeungnam University College of Medicine, Daegu, Korea; 2 Yeungnam Eye Center, Yeungnam University Hospital, Daegu, Korea; 3 Department of Ophthalmology, School of Medicine, Kyungpook National University, Daegu, Korea

**Keywords:** Cytomegalovirus retinitis, Drug dose-response relationship, Ganciclovir, Intravitreal injections, Review, Treatment outcome

## Abstract

**Background:**

Intravitreal ganciclovir is widely used to achieve effective local antiviral concentrations for cytomegalovirus (CMV) retinitis; however, to our knowledge, standardized dosing strategies have not been established, and the reported regimens vary considerably across studies. In this study, we evaluated dose-dependent treatment outcomes of intravitreal ganciclovir for CMV retinitis.

**Methods:**

The PubMed, Embase, Cochrane Library, and Scopus databases were searched through November 2025. Eligible studies included intravitreal ganciclovir monotherapy, with or without systemic antiviral therapy. Cumulative first-week intravitreal dose was calculated and classified as low dose (<4,000 µg) or high dose (≥4,000 µg). The pooled proportions for resolution, visual outcomes, recurrence, and retinal detachment were estimated using a random-effects model.

**Results:**

Eighteen studies comprising 1132 eyes were included across all outcomes. The pooled proportion of anatomical resolution was 89% (95% confidence interval, 0.77–0.95), and 74% of eyes maintained stable or improved vision. Recurrence and retinal detachment occurred in 12% and 9% of the eyes, respectively. High-dose regimens achieved a significantly higher resolution than low-dose regimens (94% vs. 73%, *p*=0.019). Visual outcomes did not differ according to dose (77% vs. 73%, *p*=0.646). Recurrence also showed no dose-dependent difference (14% vs. 8%, *p*=0.654) and was observed predominantly in patients before the introduction of highly active antiretroviral therapy. The retinal detachment rates were similar (9% vs. 10%, *p*=0.780).

**Conclusion:**

Initial intravitreal dosing at ≥4,000 µg within the first week achieved better retinitis resolution, supporting the benefit of a higher local ganciclovir concentration in the treatment of CMV retinitis.

## Introduction

Cytomegalovirus (CMV) retinitis causes significant visual loss in individuals who are immunocompromised, particularly those with advanced acquired immunodeficiency syndrome (AIDS) [1,2]. Before the introduction of highly active antiretroviral therapy (HAART), CMV retinitis affected approximately 30% of patients with AIDS, accounting for >90% of those with human immunodeficiency virus (HIV)-related blindness [3,4]. While the widespread adoption of HAART has reduced its incidence by 70% to 90% [5,6], CMV retinitis remains a major vision-threatening condition in patients who are HIV infected, especially in resource-limited settings [7].

CMV-infected eyes develop full-thickness retinal necrosis with characteristic yellow-white retinal infiltrates and associated hemorrhage [8]. If left untreated, progressive retinal destruction can result in permanent vision loss through direct tissue damage, retinal detachment, or macular involvement [9]. Beyond patients with HIV/AIDS, CMV retinitis increasingly affects other immunocompromised populations, including patients receiving hematopoietic stem cell transplants (HSCTs), solid organ transplants, and immunosuppressive therapy [10,11].

Effective treatment of CMV retinitis requires adequate drug delivery to the target tissue. Although systemic antiviral therapy with intravenous ganciclovir or oral valganciclovir provides the advantage of treating both ocular and systemic CMV disease [12], achieving adequate intraocular drug concentrations remains challenging because of the blood-retinal barrier [13,14]. Furthermore, systemic therapy carries substantial risks of myelosuppression and nephrotoxicity, which can limit treatment duration and adherence [15,16].

Intravitreal injection of ganciclovir has emerged as an alternative strategy for achieving therapeutic intraocular concentrations while minimizing systemic adverse effects [17]. However, standardized dosing guidelines are lacking. Published studies have employed widely varying dosing protocols for intravitreal ganciclovir, ranging from 0.4 mg per injection to 6 mg per injection [18-20]. While a 2-mg dose has been commonly adopted in clinical practice, some investigators have advocated higher single doses or more frequent injections to sustain therapeutic levels [21,22]. Inadequate dosing may lead to treatment failure and viral resistance, whereas excessive treatment frequency increases the burden on patients and healthcare systems, along with injection-related risks such as endophthalmitis and retinal detachment [23,24].

This meta-analysis aimed to systematically evaluate the dose-dependent outcomes of intravitreal ganciclovir therapy for CMV retinitis by comparing the efficacy and safety of different dosing regimens.

## Methods

### 1. Search strategy and study selection

A comprehensive search of PubMed, Embase, Cochrane Library, and Scopus was performed for articles from database inception to November 2025 using combinations of the following terms: (“cytomegalovirus retinitis” or “CMV retinitis”) AND (intravitreal OR intraocular) AND (ganciclovir) AND (dose* OR dosage OR microgram* OR μg OR mg). Studies were included if they (1) evaluated intravitreal ganciclovir for the treatment of CMV retinitis, (2) reported extractable data on at least one clinical outcome (i.e., resolution of retinitis, visual acuity, recurrence, or retinal detachment), and (3) included human participants who were either HIV/HAART-associated or non-HIV immunosuppressed (e.g., HSCT, chemotherapy, autoimmune disease). The exclusion criteria included studies using only systemic therapy without intravitreal treatment, studies using intravitreal agents other than ganciclovir (e.g., foscarnet, cidofovir), case reports or small case series (<five eyes), insufficient quantitative data, and reviews or abstracts.

### 2. Data extraction

After removing duplicate studies, two independent reviewers (SJ, AJ) evaluated eligible studies. The titles and abstracts were screened first, followed by a full-text review. For each study, data regarding the publication year, first author, patient immunological status (HIV/HAART vs. non-HIV immunosuppression), number of patients and eyes, and intravitreal ganciclovir dosing regimen were extracted. Total intravitreal dose administered during the first week (first-week dose) was calculated and defined as the injection dose (µg) multiplied by the number of injections performed within the first 7 days.

Studies were then classified into two groups: low-dose group (<4,000 µg during the first week) and high-dose group (≥4,000 µg during the first week). The cutoff value of 4,000 µg reflects a regimen of 2,000 µg twice weekly, which has been widely adopted in clinical practice for CMV retinitis. The outcome definitions (i.e., resolution of retinitis, visual outcomes, retinal detachment, endophthalmitis) followed those described in the original publications. Given the variability in the definitions of retinitis resolution across studies, resolution outcomes were extracted based on anatomical and clinical assessments, consistently reflecting the loss of active retinitis and cessation of lesion progression, with or without subsequent scarring.

The methodological quality of the included studies was evaluated according to Murad et al. [25], which assesses four domains: selection, ascertainment, causality, and reporting. Each study was independently assessed by two reviewers (SJ, AJ) and discrepancies were resolved by consensus.

### 3. Statistical analysis

A pooled proportion meta-analysis was conducted for each outcome. As substantial clinical and methodological heterogeneity was expected across the studies, a random-effects model using the DerSimonian–Laird method was applied to calculate pooled estimates with 95% confidence intervals (CIs). The study-level weights and between-study heterogeneity (I² statistics) were determined. Publication bias was explored by visual inspection of funnel plots.

Subgroup analyses were conducted to compare the outcomes between the low- and high-dose groups. Differences between the subgroups were evaluated using chi-square tests. All analyses were performed using the "meta" package in R (ver. 4.5.2; R Foundation for Statistical Computing, Vienna, Austria), and statistical significance was defined as *p*<0.05.

## Results

### 1. Study characteristics

**Fig. 1.**
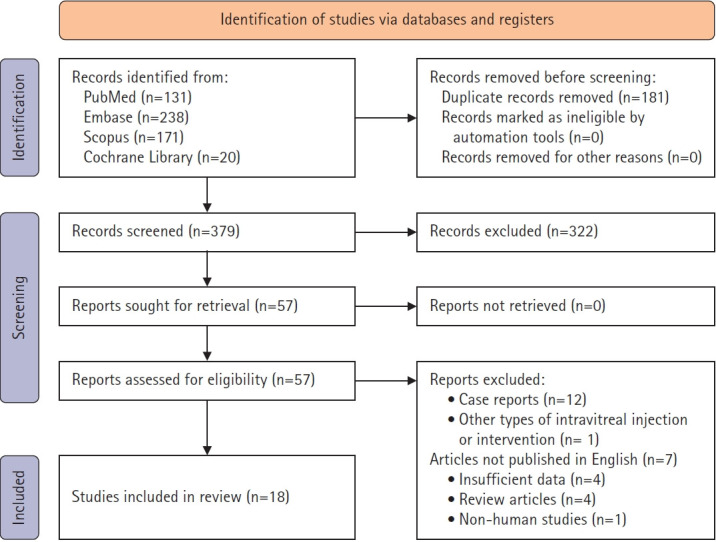
Preferred Reporting Items for Systematic Reviews and Meta-Analyses (PRISMA) flow diagram of the study selection process.

**Fig. 2.**
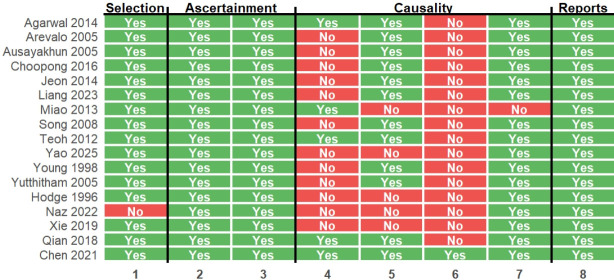
Risk of bias assessment of the included studies using the Murad criteria.

A total of 18 studies [17-21,26-38] comprising 1,132 eyes with CMV retinitis were included (Table 1). A Preferred Reporting Items for Systematic Reviews and Meta-Analyses (PRISMA) flow diagram summarizing the study selection process is shown in Fig. 1. The studies represented different immunological backgrounds, including pre-HAART and HAART-era HIV populations, as well as patients who were non-HIV immunosuppressed, such as HSCT recipients and individuals receiving chemotherapy or immunosuppressive therapy. Based on the cumulative dose of 1-week intravitreal ganciclovir, studies were categorized as either low dose (<4,000 µg) or high dose (≥4,000 µg). Among the 10 studies in the high-dose group, five achieved a high cumulative dose through twice-weekly injections of 2 to 3 mg, while the remaining five studies administered single injections of ≥4 mg per week. Based on the Murad assessment, the included studies generally demonstrated moderate-to-high methodological quality (Fig. 2).

### 2. Overall treatment outcomes

**Fig. 3.**
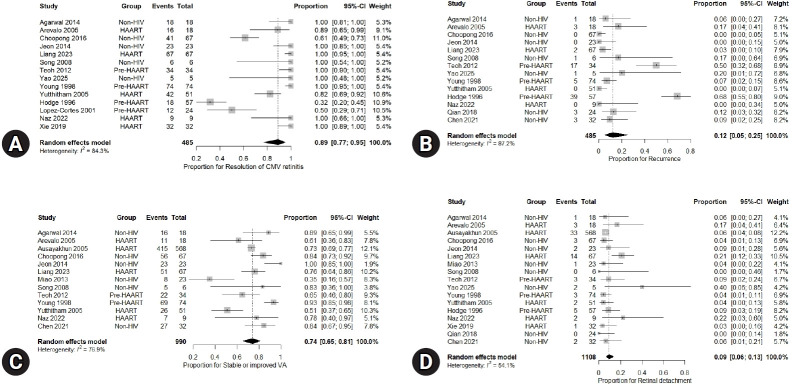
Forest plots showing pooled estimates of overall treatment outcomes of intravitreal ganciclovir for cytomegalovirus (CMV) retinitis. (A) Resolution of CMV retinitis, (B) recurrence, (C) stable or improved visual acuity (VA), and (D) retinal detachment. CI, confidence interval; HAART, highly active antiretroviral therapy; HIV, human immunodeficiency virus.

Across all studies, the pooled proportion of eyes achieving anatomical resolution was 89% (95% CI, 0.77–0.95), with considerable heterogeneity (I²=84.3%) (Fig. 3A). Recurrence was reported in 485 eyes across 14 studies. The pooled recurrence rate was 12% (95% CI, 0.05–0.25), accompanied by high heterogeneity (I²=87.2%). Two studies indicated recurrence rates of >50%, both of which were conducted in pre-HAART populations (Fig. 3B). Thirteen studies, involving 990 eyes, reported visual outcomes. The pooled proportion of eyes demonstrating improved or stable visual acuity was 74% (95% CI, 0.65–0.81; I²=76.9%) (Fig. 3C). Retinal detachment was reported in 1,108 eyes across 17 studies. The pooled proportion of detachment was 9% (95% CI, 0.06–0.13) with moderate heterogeneity (I²=54.1%) (Fig. 3D).

### 3. Dose-response analysis based on the first-week intravitreal dose

**Fig. 4.**
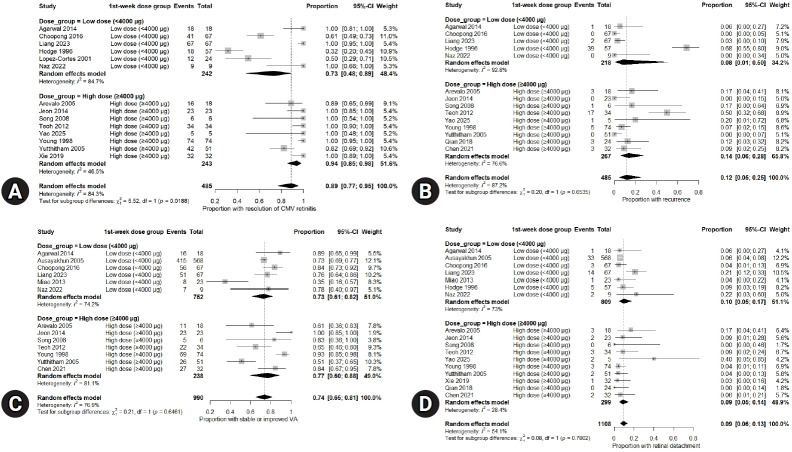
Forest plots showing pooled estimates of cytomegalovirus (CMV) retinitis outcomes according to cumulative first-week intravitreal ganciclovir dose (<4,000 µg vs. ≥4,000 µg). (A) Resolution of CMV retinitis, (B) recurrence, (C) stable or improved visual acuity (VA), and (D) retinal detachment. CI, confidence interval; HAART, highly active antiretroviral therapy; HIV, human immunodeficiency virus.

A comparison of retinitis resolution according to the cumulative first-week intravitreal dose is shown in Fig. 4A. Studies administering ≥4,000 µg in the first week demonstrated a higher pooled resolution rate than those using <4,000 µg (94% vs. 73%, subgroup difference *p*=0.019) (Fig. 4A).

Recurrence rates did not differ significantly according to dose category. The pooled recurrence rates were 14% and 8% in the high-dose and low-dose groups, respectively (*p*=0.654) (Fig. 4B).

Visual outcomes did not show a statistically significant dose-dependent pattern. The pooled proportion of eyes with improved or stable visual acuity was similar between the two groups (77% vs. 73%, *p*=0.646) (Fig. 4C). Retinal detachment rates were low for both dosing strategies with no significant between-group difference (9% vs. 10%, *p*=0.780) (Fig. 4D).

## Discussion

This meta-analysis evaluated dose-dependent outcomes of intravitreal ganciclovir therapy for CMV retinitis. Our results demonstrated that higher induction doses achieved significantly better retinitis resolution rates than lower doses, suggesting the necessity for adequate and continuous drug concentrations in local ganciclovir therapy. Visual outcomes were similar to those reported in the literature, with 74% of the patients achieving stable or improved vision. Recurrence rates did not differ significantly between the dose groups but were markedly influenced by systemic immune status, with substantially higher recurrence in patients not receiving HAART.

The high resolution rates of CMV retinitis observed in our study align with previous meta-analyses that reported approximately 90% resolution with intravitreal ganciclovir therapy [39]. This confirms that intravitreal antiviral treatment can effectively suppress retinitis in most patients. The pharmacokinetic advantages of intravitreal injections explain these favorable outcomes; direct intraocular delivery achieves therapeutic concentrations exceeding those possible with systemic administration that struggles to cross the blood-retinal barrier effectively [13,40]. Studies have shown that vitreous concentrations following intravitreal ganciclovir injection remain above the 50% inhibitory concentration for CMV for approximately 1 week [41], providing sustained viral suppression.

The visual outcomes in our cohort showed that 74% of the patients maintained stable or improved vision, which is consistent with prior reports. Young et al. demonstrated that 85% of patients receiving high-dose intravitreal ganciclovir maintained a visual acuity of 20/40 or better compared to 59% receiving intravenous therapy alone [17]. Similarly, other studies have indicated visual stability or improvement in 65% to 88% of treated eyes [35,42].

The superior resolution rates in the high-dose group provide evidence of a dose-response relationship in intravitreal ganciclovir therapy. In our dose-response analysis, regimens achieving a cumulative first-week dose of ≥4,000 µg showed a higher pooled resolution rate than those using lower doses (94% vs. 73%, *p*=0.019). This finding has important clinical implications for treatment frequency because many previous studies have adopted a dose of 2.0 mg intravitreal ganciclovir per injection [17,43]. Indeed, among the 10 studies included in our high-dose group, five achieved weekly cumulative doses of ≥4.0 mg through twice-weekly injections of 2.0 to 3.0 mg.

Visual outcomes did not differ significantly between the high- and low-dose regimens (77% vs. 73%, *p*=0.646). Previous studies have shown that the degree of visual improvement remains modest, even with successful resolution. The causes of visual deterioration include retinal necrosis, optic nerve involvement, cystoid macular edema, and retinal detachment [4,44]. Our findings are consistent with those of previous studies, suggesting that visual recovery is limited by the irreversible structural damage and complications of CMV retinitis [3,45]. Retinal detachment, a major sight-threatening complication, occurred in approximately 10% of the eyes in our meta-analysis and showed moderate heterogeneity across studies.

A critical question raised by our findings is whether the benefits of high-dose treatment are derived from an increased injection frequency or higher concentrations per injection. In our analysis, half of the high-dose groups achieved higher cumulative doses by increasing the injection frequency (twice weekly) rather than by increasing the dose per injection. Chen et al. [21] compared single high-dose injections (6,000 µg once weekly) with twice-weekly moderate-dose injections (3,000 µg twice weekly) during the induction phase. Both groups achieved 100% resolution, as measured by CMV DNA polymerase chain reaction. However, the high-dose once-weekly group demonstrated a slightly shorter time to resolution (21 days vs. 26 days, *p*=0.282) and required significantly fewer total injections (*p*=0.016). In contrast, doses of ≥4 mg/0.4 mL have been associated with crystallization and potential retinal toxicity in previous case reports and experimental studies [46,47]. Although these results indicate that single high-dose administration may be a viable alternative to frequent standard-dose injections, further studies are required to establish optimal dosing strategies that balance efficacy and safety.

The recurrence rates in our analysis did not differ significantly between the high- and low-dose groups. Rather, recurrence was more frequently observed in patients not receiving HAART. Large prospective studies in patients with AIDS have demonstrated that the rate of retinitis progression is substantially lower in patients receiving HAART who are immune-recovered (approximately 0.1–0.2 per person-year) than in patients who are immunocompromised (approximately 2–3 per person-year) [48]. These findings suggest that the long-term suppression of CMV retinitis depends more on the host’s immune status than on the intensity of the initial local treatment. As CMV is a latent virus, inadequate immune function facilitates viral reactivation despite local antiviral therapy [49].

Several limitations should be considered when interpreting the results. First, owing to the rarity of CMV retinitis and the lack of clear dosing guidelines for intravitreal ganciclovir therapy, well-designed randomized controlled trials are scarce. As a result, our meta-analysis predominantly consisted of case series that were inherently susceptible to selection bias and residual confounding. Second, the follow-up duration varied substantially across studies, ranging from weeks to months, potentially affecting the recurrence rate estimates and long-term outcome assessments. Third, considerable heterogeneity existed in patient characteristics, particularly regarding systemic immune status and concurrent treatments. Despite these limitations, to the best of our knowledge, our study has significant clinical value as the first systematic review and meta-analysis to specifically examine the dose-dependent outcomes of intravitreal ganciclovir monotherapy for CMV retinitis, excluding other agents such as foscarnet and cidofovir. Future prospective randomized controlled trials with standardized dosing regimens, uniform follow-up protocols, and detailed documentation of immune status are warranted to validate these findings and establish evidence-based treatment guidelines for intravitreal ganciclovir therapy for CMV retinitis.

In conclusion, our study demonstrates that higher cumulative doses of intravitreal antiviral therapy improve resolution rates in CMV retinitis, whereas recurrence prevention depends on the systemic immune status. From a practical clinical perspective, when using the standard 2 mg dose per injection, our findings suggest setting the initial injection frequency to twice weekly or more with long-term monitoring of the patient’s immune status. Given prior reports of ganciclovir crystallization or retinal toxicity at very high concentrations, caution is warranted when attempting to achieve higher doses using single high-dose injections. Future large-scale randomized clinical trials are needed to verify these findings and determine the optimal dose and injection schedule to improve visual outcomes and minimize recurrence in patients with CMV retinitis.

## Figures and Tables

**Table 1.** Treatment protocols of included studies

No.	Study	Year	Patient group	No. of patients	No. of eyes	IVG dose (μg)	Interval	First-week dose (μg)	Four-week cumulative dose (μg)	Systemic therapy
1	Agarwal et al. [26]	2014	Non-HIV	10	18	2,000	Weekly	2,000	8,000	Oral valganciclovir (partial)
2	Arevalo et al. [27]	2005	HAART	13	18	5,000	Weekly	5,000	20,000	IV and/or oral ganciclovir
3	Ausayakhun et al. [28]	2005	HAART	363	568	2,000	Weekly, followed by every 2–4 weeks	2,000	8,000	IV and/or oral ganciclovir
4	Choopong et al. [19]	2016	Non-HIV	49	67	2,000	Weekly, followed by every 2 weeks	2,000	8,000	IV ganciclovir (partial)
5	Jeon and Lee [30]	2015	Non-HIV	15	23	2,000	Twice weekly, followed by weekly	4,000	12,000	IV → oral valganciclovir
6	Liang et al. [18]	2023	HAART	50	67	400 or 1,000	Weekly	400 or 1,000	1,600 or 4,000	IV → oral valganciclovir
7	Miao et al. [32]	2013	Non-HIV	14	23	1,000	Weekly for 4 weeks	1,000	4,000	None
8	Song et al. [34]	2008	Non-HIV	3	6	2,000	Twice weekly, followed by weekly	4,000	12,000	IV ganciclovir (partial)
9	Teoh et al. [35]	2012	Pre-HAART	24	34	2,000	Twice weekly, followed by weekly	4,000	16,000	None
10	Yao et al. [37]	2025	Non-HIV	3	5	6,000	Weekly for 2–3 weeks	6,000	12,000	Letermovir or maribavir
11	Young et al. [17]	1998	Pre-HAART	42	74	2,000	Twice weekly, followed by weekly	4,000	14,000	IV ganciclovir
12	Yutthitham and Ruamviboonsuk [38]	2005	HAART	33	51	4,000	Every 2 weeks	4,000	8,000	None
13	Hodge et al. [29]	1996	Pre-HAART	40	57	400	Weekly	400	1,600	IV ganciclovir
14	Lopez-Cortes et al. [31]	2001	Pre-HAART	17	24	400	Weekly	400	1,600	IV ganciclovir/foscarnet
15	Naz et al. [33]	2022	HAART	5	9	2,000	Weekly for 3 weeks	2,000	6,000	Oral valganciclovir
16	Xie et al. [36]	2019	HAART	23	32	3,000	Twice weekly	6,000	15,000	IV → oral valganciclovir
17	Qian et al. [20]	2018	Non-HIV	24	24	6,000	Weekly	6,000	16,500	None
18	Chen et al. [21]	2021	Non-HIV	22	32	6,000	Weekly	6,000	24,000	None

IVG, intravitreal ganciclovir; HIV, human immunodeficiency virus; HAART, highly active antiretroviral therapy; IV, intravenous; PO, oral administration.
